# Preferences for ARV-based HIV prevention methods among men and women, adolescent girls and female sex workers in Gauteng Province, South Africa: a protocol for a discrete choice experiment

**DOI:** 10.1136/bmjopen-2015-010682

**Published:** 2016-06-27

**Authors:** Matthew Quaife, Robyn Eakle, Maria Cabrera, Peter Vickerman, Motlalepule Tsepe, Fiona Cianci, Sinead Delany-Moretlwe, Fern Terris-Prestholt

**Affiliations:** 1 Department of Global Health and Development, London School of Hygiene and Tropical Medicine, London, UK; 2 Wits RHI, University of the Witwatersrand, Johannesburg, South Africa; 3 School of Social and Community Medicine, University of Bristol, Bristol, UK; 4 Progressus Research and Development, Johannesburg, South Africa; 5 Department of Public Health East, Dr Steeven's Hospital, Dublin, Republic of Ireland

**Keywords:** HEALTH ECONOMICS, PUBLIC HEALTH

## Abstract

**Introduction:**

For the past few decades, condoms have been the main method of HIV prevention. Recent advances in antiretroviral (ARV)-based prevention products have substantially changed the prevention landscape, yet little is known about how popular these products will be among potential users, or whether new methods might be used in conjunction with, or instead of, condoms. This study will use a discrete choice experiment (DCE) to (1) explore potential users' preferences regarding HIV prevention products, (2) quantify the importance of product attributes and (3) predict the uptake of products to inform estimates of their potential impact on the HIV epidemic in South Africa. We consider preferences for oral pre-exposure prophylaxis; a vaginal microbicide gel; a long-acting vaginal ring; a SILCS diaphragm used in concert with gel; and a long-acting ARV-based injectable.

**Methods and analysis:**

This study will gather data from 4 populations: 200 women, 200 men, 200 adolescent girls (aged 16–17 years) and 200 female sex workers. The DCE attributes and design will be developed through a literature review, supplemented by a thematic analysis of qualitative focus group discussions. Extensive piloting will be carried out in each population through semistructured interviews. The final survey will be conducted using computer tablets via a household sample (for women, men and adolescents) and respondent-driven sampling (for female sex workers), and DCE data analysed using a range of multinomial logit models.

**Ethics and dissemination:**

This study has been approved by the University of the Witwatersrand Human Research Ethics Committee and the Research Ethics Committee at the London School of Hygiene and Tropical Medicine. Findings will be presented to international conferences and peer-reviewed journals. Meetings will be held with opinion leaders in South Africa, while results will be disseminated to participants in Ekurhuleni through a public meeting or newsletter.

**Strengths and limitations of this study**

- This study uses a novel discrete choice experiment (DCE) to elicit people's preferences for new antiretroviral (ARV)-based HIV prevention products.
- The results of the DCE will allow us to explore how respondents value different product characteristics. We will also be able to predict whether products will be used alongside, or instead of condoms, a critical element of assessing their potential impact.
- We will draw out policy recommendations from our findings, in particular informing mathematical models to evaluate the impact and cost-effectiveness of a range of new prevention products.
- The DCE choice tasks are hypothetical in nature and will be carried out in Ekurhuleni Metropolitan Municipality, near Johannesburg, South Africa. The results may not be generalisable to other settings. Female sex workers will be recruited through respondent-driven sampling, rather than a randomised method.

## Introduction

Despite intense efforts to reduce HIV incidence, its estimated prevalence remains high in South Africa. The fourth national population-based survey conducted by the Human Sciences Research Council, estimated prevalence to be 12.6% in 2012, an increase on the previous survey estimate of 10.9% in 2008.^1^ This increase may be explained partly by expanded access to antiretroviral (ARV) treatments and associated reductions in mortality, however, there is also evidence of continued sexual transmission of HIV in those aged 15 years or older. HIV infection is not borne equally in the South African population; women are 1.4 times more likely to be living with HIV compared to men, while adolescent girls (aged 15–19 years) are at 8 times greater risk of being HIV positive than boys of the same age.^1^ Female sex workers (FSWs) are designated a key population for HIV treatment and prevention activities, and are around four times more likely to be living with HIV than South African women of reproductive age from the general population.^2^ ^3^

The HIV prevention landscape continues to shift substantially, not least due to emerging evidence that ARV drugs can be used for HIV prevention. The HPTN 052 trial demonstrated the ability of ARVs to reduce the infectiousness of HIV positive persons through suppressing viral loads, and led to the development of treatment-as-prevention programming.^4^ Furthermore, ARV-based oral pre-exposure prophylaxis (PrEP) has been shown to offer a high degree of protection from HIV acquisition in different populations and contexts worldwide.^5–9^ In 2012, WHO recommended oral PrEP for use in specific ‘high-risk’ populations, such as serodiscordant couples. This recommendation was broadened in September 2015 with PrEP recommended for any person at ‘substantial risk’ of HIV acquisition, and not necessarily restricted to those in key populations.^10^

Despite the success and subsequent licensure of oral PrEP, there is an increasing recognition that the characteristics of effective prevention options may vary across different population groups. It is important that prevention options are tailored to fit well with the lifestyles of potential users, and recent efforts have focused on developing novel methods of delivering ARV drugs for HIV prevention. Outside of oral PrEP, there are numerous products in various stages of development including: vaginal microbicide gels used daily or at every sex act; long-acting vaginal rings; a SILCS diaphragm used in concert with gel; and long-acting injectable products.^5^ ^6^ ^8^ ^11–13^

Problems with adherence have plagued clinical trials of shorter-acting products such as microbicide gels, highlighting the need for methods that are attractive and easy for people to use.^6^ ^9^ The development of longer acting products, such as the monthly applied vaginal ring or the three-monthly injection, has the potential to increase adherence, uptake and thus effective coverage.^14^ ^15^ However, until products have been developed and rolled out to a population, it is difficult to accurately predict whether or how much they will be used, or whether they will be used in addition to, or instead of condoms which offer multipurpose protection.^16^

Attention has also turned to the potential for new products to meet the broader reproductive and sexual health needs of many individuals. These needs are not limited to protection against HIV and other sexually transmitted infections (STIs), but also access to safe and reliable contraceptives. Currently, the only products which protect against HIV, STIs and unwanted pregnancies are the male and female condoms, and so there may be demand for additional product choices which confer combination protection. As such, considerable research has focused on developing multipurpose technologies (MPTs) which offer protection against HIV, STIs and pregnancy.^17^ Efficacy trials for MPTs are planned and products could be made available on the market in the next decade.^11^ ^14^

In the context of these advances in the field of HIV prevention, it is important to identify and explore the determinants of demand for new technologies. Understanding user preferences can not only assist prevention efforts by predicting product uptake, but also help refine the development of new products. We plan to undertake a study that will use a discrete choice experiment (DCE) to quantify potential users' stated preferences of ARV-based prevention products in a South African setting, predict uptake of new products, and assess the extent to which condom use might be reduced following their introduction.

DCEs are, theoretically, robust economic tools, and can be particularly informative when there are little or no data on observed behaviour.^18–20^ DCEs ask people to choose between a number of hypothetical scenarios, where each choice is described by a set of attributes. By assessing how choices vary according to different attribute levels, researchers are able to assess what is important to people as they choose. Furthermore, DCEs allow researchers to quantitatively elicit the key drivers of individuals' decisions, and can predict future behaviour.^21^ DCE methods have been used extensively in fields of applied economics, particularly transport and environmental economics.^22–24^ In health, they have been applied across a range of disease prevention areas including voluntary medical male circumcision, vaccination, STI treatment and contraception.^25–28^

A particularly novel use of this research will be for subsequent work to integrate DCE-derived uptake predictions from this study within an infectious disease-modelling framework to estimate the potential impact and cost-effectiveness of introducing new HIV prevention products. Such models often rely on assumed levels of uptake based on expert opinion, or uptake of similar interventions.^29^ These assumptions are frequently not based on data, and so are likely to produce inaccurate projections. This study will build on previous research suggesting that DCEs may provide more data-driven, dynamic and realistic estimates of product uptake in the absence of observed data.^30^

## Methods and analysis

### Overview of approach and methods

This study will build on previous mixed-methods research to explore consumer preferences for HIV prevention products in South Africa. Primary formative research will be carried out among target groups before the implementation of a survey with 800 participants: 200 men and 200 women aged between 18 and 45 years, randomly sampled from the general population, 200 adolescent girls (aged between 16 and 17 years), also randomly sampled from the general population, and 200 FSWs sampled using respondent-driven sampling, a common approach for collecting data from hard-to-reach populations.^31^

The DCE will be carried out among all self-reported HIV-negative persons sampled. Although self-reported HIV status is not necessarily a reliable indicator of serostatus, it can still be useful for hypothetical DCE surveys, assuming that respondents answer according to their perceived HIV status. In an effort to maximise reporting accuracy, we will use an experienced team of interviewers with additional training, focusing on making participants comfortable, and reinforcing confidentiality throughout the interview.

A 2005 study in the same municipality used qualitative focus group discussions (FGDs) and individual interviews to develop a DCE on a set of HIV prevention products under development at the time.^29^ The research identified which product characteristics (or attributes) consumers valued, how the levels of these might vary, and optimal ways to present these to participants in a choice experiment. To develop relevant and meaningful choice tasks, the current study will build on this previous formative research, alongside an updated review of the literature, and intensive piloting with each population.

In a DCE, respondents are presented with a number of options which are each described by attributes of particular levels. Respondents are first asked to choose their most favoured option from two or more alternatives, and then they continue with this process which is repeated over a number of different choice sets. Attribute levels are systematically varied between sets according to an experimental design which aims to maximise the statistical efficiency of data collection. For each choice set, it is assumed that respondents choose the scenario which would give them the most benefit, and choices are, therefore, indicative of an underlying utility function. Econometric analysis of DCE data estimates the utility functions of respondents which quantitatively weights the value placed on each attribute. A more detailed analysis is possible through the inclusion of sociodemographic or other information as explanatory variables in these functions.^32^

**Figure 1:**
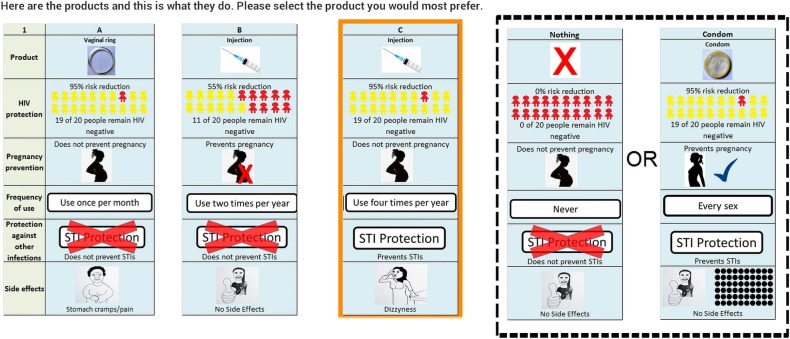
Example of a discrete choice experiment choice task to elicit HIV prevention product preferences from HIV negative women.

DCEs give a quantitative indication of the strength of an individual's preference for one attribute (such as efficacy in preventing HIV infection) relative to another (such as frequency of application). Results from DCEs are conducive to directly informing policy by allowing inference of the key drivers of individual behaviour in responding to different policy environments, and enable the simulation of how choices might change under different circumstances. An example of a DCE is shown in figure 1, where figure 1A represents a vaginal ring, and figure 1B and 1C different injectable products. The opt-out alternative in the final column will be presented as what a respondent reported using in their last sexual encounter, that is, the attributes of a condom if the participant used a condom, or the characteristics of using no protection otherwise. This is to allow the estimation of unconditional demand for new products.

**Figure 2:**
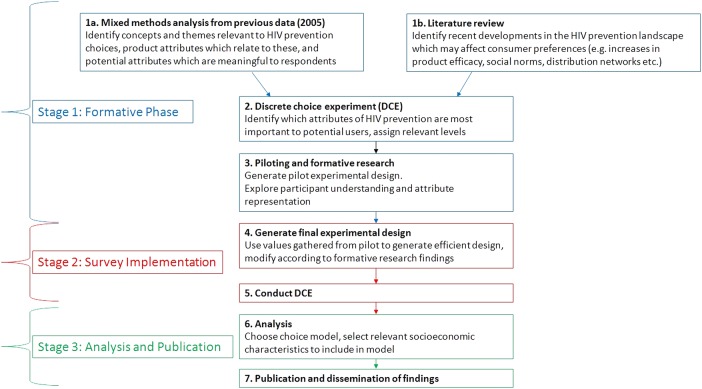
Study design. DCE, discrete choice experiment.

The study design is shown in figure 2, and consists of three stages: an extensive formative phase (including generating an initial design, piloting and pretesting of the DCE), implementation of the DCE and data analysis and publication. The DCE design began in July 2015 and piloting in September 2015. Data collection for the final DCE is expected to be carried out between October and December 2015. This study will conform to the best-practice guidelines of the International Society for Pharmacoeconomics and Outcomes Research Guidelines for Good Research Practices for conjoint analysis in health.^33^

### Stage 1: formative phase—development and piloting of choice tasks, attributes and levels

A thematic analysis of qualitative data gathered in an earlier study^29^ led to the identification of key attributes which were important to the respondents' choice of a HIV prevention product. These will be explored in the context of more recent literature to account for evolution in the HIV prevention field, for example, changes in technology (eg, product efficacy) and policy (eg, potential for free provision by the South African Government). Then, between 5 and 10 ‘pre-pilot’ interviews will be carried out in each population to assess participants' understanding of background questions and DCE choice tasks, explore the most meaningful representations of attributes and levels, and identify any issues with the tablet-based implementation of the DCE. The pre-pilot stage will be critical in creating clear, relevant and realistic choice sets which are presented to participants in an understandable manner. The DCE to be piloted is unlabelled, meaning all products will not be presented at once, but each will appear as an available alternative according to the experimental design. The six attributes of the DCE and their levels are shown in table 1.

**Table 1:** Attributes and levels

Attribute	Levels
Product	Oral pre-exposure prophylaxis, microbicide gel, microbicide gel with SILCS diaphragm, vaginal ring, injectable pre-exposure prophylaxis
HIV protection	55%, 75%, 95%
Pregnancy prevention	Prevents pregnancy, does not prevent pregnancy
Frequency of use*	Every time you have sex, once per day, once per week, once per month, once per 3 months, once per 6 months, once per year
Protection against other STIs	Protects against other STIs, does not protect against other STIs
Side effects (probability of occurrence fixed)	Stomach cramps/pain, nausea/feeling sick, dizziness, none

*As no product can be used at all frequencies, the design will contain constraint terms where only relevant frequencies will be presented alongside products. Frequencies were chosen to be informative for product development: oral PrEP—daily, weekly, monthly; microbicide gel—coitally; SILCS diaphragm and microbicide gel—coitally, daily; vaginal ring—weekly, monthly, three-monthly, six-monthly; injection—three-monthly, six-monthly, annually.

SILCS; STI, sexually transmitted infections.

We will include five new prevention products, chosen to reflect the range of potential ARV delivery mechanisms. Some (eg, oral PrEP) are fully developed and are in the later stages of global licensing, while others (eg, injectables) are a few years from efficacy trials and potential roll-out. Women will be presented with the full range of products, while men will only be presented with male-initiated options (ie, oral PrEP and injectables). We include two products based on microbicide gels, despite recent unsuccessful effectiveness trials.^6^ ^9^ Trial results suggest that gels were efficacious when used adherently, however, many participants were not able to use the products consistently enough as part of everyday life. Including these products in the DCE will allow us to explore how preferences for products vary by characteristics, such as frequency of use and potential side effects, and provide an opportunity to compare DCE predictions to reality. We note that common side effects were chosen through reports from PrEP trials and ART regimens using ARV drugs.^34^ ^35^

FGDs will be carried out with FSWs, as the risk factors and sexual relationships of this group are likely to be substantively different from other population, while it was noted that there was a gap in the literature and in local knowledge on FSW preferences. It is a limitation of this study that we are unable to carry out FGDs with men or adolescent women; however, we will use the expertise and experience of local collaborators alongside identifying substantive literature to assess relevant attributes for these groups. In the FSW FGD, themes related to HIV prevention choices and negotiation of protection with clients and partners will be explored in four FGDs with between 8 and 12 participants in each. The qualitative data from the focus groups will then be analysed using thematic analysis to inform the final version of the DCE survey tools.^36^ These discussions will provide novel insights into decision-making by FSWs around HIV prevention which have never been explored in a DCE context before.

The DCE will be piloted through an initial fractional factorial design which will be generated by specialist NGENE software^37^ and tested face to face with a subsample of 20 respondents, five from each population group (men, women, adolescent girls and FSWs). These pilots will aim to assess how well respondents understand questions and responses, whether tablet-based enumeration is feasible and reliable, and explore different presentations of DCE tasks and attributes. Furthermore, the responses from these DCE tasks will be analysed using a multinomial logit model (MNL) to obtain point estimates of utility function parameters. These estimates will be used in generating a statistically efficient design for the final DCE.

The attributes and levels shown above yield 1260 possible product profiles, far too great a number to present to all participants. Recent advances in DCE design have led to the development of ‘efficient’ designs which, when informed by prior information from a pilot study and/or the literature, offer more reliable parameter estimates when compared with traditional orthogonal designs. To ensure realistic choice data and avoid overestimating demand for new products,^32^ participants can opt out of choosing a new product with a fourth alternative presented of ‘do what I did last time’ (ie, use a condom or nothing). Since an unlabelled design is used to reduce the number of choices within a set from six to four, it is likely (and certain in the male DCE) that some products will be presented twice in the same choice set, with different attribute levels. Interviewers will be fully trained to explain this nuance to participants.

Finally, data will be captured in the final questionnaire on salient background characteristics of participants including: age, gender, quality of life, reproductive history, relationship history and HIV knowledge. This data will allow the analysis of how preferences are shaped by life circumstances, for example, if a respondent is seeking to conceive with a partner. Through framing choice tasks around the last sex act, we will be able to explore how preferences may vary by partner type, for example, between non-commercial and commercial partners of FSWs, or between long-term and short-term sexual relationships. Data will also be gathered on the gender of respondents' most recent sexual partner. The piloting process will inform which characteristics should be included in final data analysis as explanatory variables for preferences in HIV prevention products. Finally, we note that while trials of some products (oral PrEP and a vaginal ring) will be ongoing in some populations in South Africa during this study,^38^ the geographically concentrated and research-naive populations chosen for this work are very unlikely to have experience of using these (eg, through participation in trials). We will record if participants have prior experience of using any of these products.

### Stage 2: administration of DCE

#### Participants and recruitment

The survey and DCE will be administered using Open Data Kit (https://opendatakit.org/) software on tablet computers. The acceptability and feasibility of tablet-based data collection will be assessed during the formative stage, although it is expected that it will minimise missing data, and reduce laborious data entry and cleaning.^39^ ^40^ Participants will be given a ZAR 50 (GBP £2.50) voucher as compensation for their time. Participants will be asked to self-report their HIV status, and we aim to maximise accuracy in reporting through careful confidentiality and sensitivity training of the interviewer team, which has considerable experience in collection of HIV and sexual health data.

To ensure that choices are relevant and meaningful to participants, three steps will be taken to maximise the realism of choice scenarios. First, all interviewers will be equipped with a full set of example products: a real SILCS diaphragm, alongside placebo PrEP tablets (similarly coloured, shaped and sized), vaginal rings, microbicide gels and injections (an empty syringe). Participants will be encouraged to touch and explore the products as much as they wish before beginning choice tasks. Second, interviewers will be thoroughly trained and tested on how to describe products to participants. In addition, concise and clear information sheets will be used when explaining the tasks, products and attributes. Third, the statistical design of the DCE will be such that only relevant products are presented to different groups, and relevant attributes for different products. For example, only injectable and PrEP options will be presented to men, while the frequency of injections will be restricted to between once per month and once per year.

If participants choose an ARV-based, non-condom product, they will subsequently be asked whether or not, if the product was available, they would have used it the last time they had sex. If they indicate that they would, they will be asked whether they would have used it alongside, or instead of, a condom. This will enable us to ascertain whether new products will be used in combination with, or in substitute of, condoms. Since there is likely to be no additional benefit from dual use of ARV-based products, nor would medical advice suggest this would be something users should do, the DCE framework does not allow for the combination use of ARV-based products, except the SILCS diaphragm which is used in concert with gel.

Primary data will be collected in Ekhurhuleni Metropolitan Municipality, Gauteng Province. The Municipality was selected as the study site because it contains a broad range of residential contexts, representing a range of demographic, socioeconomic and cultural characteristics. We employ a proportional cluster sampling strategy, stratified by population. For the 200 men and 200 women, the nature of the household sampling method means that we may be able to interview both cohabiting partners in a relationship; assuming a sufficient number of individuals in cohabiting relationships are available and consent to participate, analysis of preferences within relationships would be a particularly novel element of this research.

Different enumeration teams will interview respondents from each group. For the general population, a specialist local data collection firm with over 20 years of experience, Progressus Research and Development (http://www.progressus.co.za/), will manage the survey process, generate the sampling design, and carry out the DCE. On finding an adult present in a household, the interviewer will identify him/herself, explain the study, and request permission to note down all women, men and adolescent girls living in the house ordered by age. We expect the whole survey, including DCE, will take around 30–45 min to complete.

Two hundred FSWs will be recruited through RDS. We will employ peer educators who will first locate sex work ‘hotspots’ in the Vosloorus area, before selecting 7–10 seeds among FSWs operating in different areas such that different social networks are reached (eg, FSWs working in brothels, on the street or in bars or taverns). These women will be invited to participate in the study and given a ZAR 50 (GBP £2.50) voucher as compensation for their time, before receiving three coupons containing the study information to distribute to colleagues. When one of these colleagues attend the study site, both they and their recruiter receive a small incentive in the form of a ZAR 20 (GBP £1) voucher. The amount of compensation was based on similar studies among FSWs in South Africa, and is designed to be high enough to compensate for potential loss of one client during study participation, but not so high as to encourage significant fraudulent enrolment.

#### Sample size

The DCE literature has not yet reached consensus on the best way to successfully estimate the sample size required in a DCE study to return meaningful, statistically robust parameter estimates. Applying the popular rule of thumb of Johnson and Orme,^41–43^ we estimate that a sample size of 90 should be sufficient to estimate parameters of the DCE structure shown in figure 1. Similarly, the literature suggests that between 20 and 30 observations per choice set can provide precise parameter estimates.^18^ ^44^ This indicates that our sample size of 800 will be sufficient to estimate parameters over the entire population, as well as to explore any variations in preferences which might exist between groups.

### Stage 3: data analysis

Results from this study will aid future development and rollout of HIV prevention products by identifying key product attributes likely to influence an individual's decision to use such products. Although there are a number of ways to analyse DCE data, the literature generally advises to start with a simple MNL model, and progressively explore other model specifications.^32^ A notable restriction of the MNL model is that it assumes the ‘irrelevance of independent alternatives’ (IIA), specifically that the odds of choosing one class over another does not depend on the wider set of alternatives. This is often not realistic, and we will employ discrete choice models such as the mixed MNL (MMNL) and generalised MNL (GMNL) models. These relax some assumptions of the MNL model (including the IIA restriction) as model coefficients are allowed to vary over individuals through the inclusion of stochastic components. Choice data from this study will be analysed through MNL, MMNL, nested logit and generalised MMNL models to fully explore heterogeneity in the data.^32^

Interactions between DCE attributes and respondent characteristics (such as the interaction between perceived HIV risk and preferences for product efficacy) will be explored by their inclusion as explanatory variables when estimating utility functions. Model results will be presented as parameter estimates with SEs. The marginal rate of substitution between attributes will also be calculated.

Finally, the RDS sample among FSWs will be analysed with consideration of the non-random, chain-referral nature of the data through mathematical weighting to compensate for non-random participant recruitment. RDS data require a number of assumptions to hold for statistical inferences to be valid; however, when attempting to reach hidden populations such as FSWs, RDS is an increasingly popular and reasonably robust method.^31^ ^45^

## Ethics and dissemination

### Ethical considerations

This study has been reviewed and approved by the University of the Witwatersrand Human Research Ethics Committee and the Research Ethics Committee at the London School of Hygiene and Tropical Medicine. All participation in the DCE, alongside supporting qualitative studies will be voluntary and subject to completion of written informed consent. When interviewing adults in households where adolescent girls aged 16–17 years are present, interviewers will ask for assent from the young woman, aim to obtain guardian consent, and interview the adolescent. The informed consent processes will be administered in private (including from a parent or guardian of an adolescent subject). A comprehensive distress protocol will ensure that participants who reveal distressing or harmful events during the survey will be referred to named persons at local clinics and NGOs.

All information provided by respondents will be kept secure and confidential. Paper-based informed consent forms and household survey frames will be kept separately from questionnaire data to protect the identity of the participants. Participants do not need to use their real names in any of the interview formats, while the background survey will not collect any identifiable information from respondents outside of salient socioeconomic and sexual history characteristics. It will be made clear to all participants that they have the right to withdraw from the research at any point in time. Participants will be informed that there is no immediate benefit to them for taking part in the study, but that the information they give can help develop future products and services. Participants will receive a ZAR 50 (£2.50) voucher as a token of appreciation for their time.

## Dissemination

Results will be published through peer-reviewed journals and via national and international conference presentations. Meetings will be held with opinion leaders in South Africa and organisations working in the area of HIV prevention. Results will be disseminated to participants in Ekurhuleni through a public meeting or newsletter; extra care will be taken here to ensure participant anonymity.
